# Therapeutic potential of *Clostridium butyricum* anticancer effects in colorectal cancer

**DOI:** 10.1080/19490976.2023.2186114

**Published:** 2023-03-20

**Authors:** Hui Xu, Haidan Luo, Jiayu Zhang, Kai Li, Mong-Hong Lee

**Affiliations:** Guangdong Research Institute of Gastroenterology, and Guangdong Provincial Key Laboratory of Colorectal and Pelvic Floor Disease, The Sixth Affiliated Hospital of Sun Yat-sen University, Guangzhou, China

**Keywords:** *Clostridium butyricum*, MYC, ubiquitination, TYMS, 5-FU, immunotherapy, Anti-PD1

## Abstract

Probiotic roles of *Clostridium butyricum* (C.B) are involved in regulating disease and cancers, yet the mechanistic basis for these regulatory roles remains largely unknown. Here, we demonstrate that C.B reprograms the proliferation, migration, stemness, and tumor growth in CRC by regulating pivotal signal molecules including MYC. Destabilization of MYC by C.B supplementation suppresses cancer cell proliferation/metastasis, sensitizes 5-FU treatment, and boosts responsiveness of anti-PD1 therapy. MYC is a transcriptional regulator of Thymidylate synthase (TYMS), a key target of the 5-FU. Also MYC is known to impact on PD-1 expression. Mechanistically, C.B treatment of CRC cells results in MYC degradation by enhancing proteasome-mediated ubiquitination, thereby mitigating MYC-mediated 5-FU resistance and boosting anti-PD1 immunotherapeutic efficacy. Together, our findings uncover previously unappreciated links between C.B and CRC cell signaling, providing insight into the tumorigenesis modulating mechanisms of C.B in boosting chemo/immune therapies.

## Introduction

Colorectal cancer (CRC) is one of the deadliest cancer types with strong resistance to various therapies,^1^ and is a cancer type with a very high mortality rate^2^. Therefore, it is pivotal to characterize potential risk factors, cancer biomarkers^3^, and microbiome markers^4–6^ to achieve effective treatment for CRC. The intestinal tract microbiota, containing at least 38 trillion bacteria, is critical for the maintenance of homeostasis and health^7^. Colon is a location with the largest number of gut microbes linking to CRC,^5,8,9^. The important advances in microbiome studies have characterized the critical protecting role of intestinal microbes, such as probiotics, in human cancers. Probiotic bacteria impact on physiological and immunological mechanisms; therefore, they may have antitumor activities. *Bifidobacterium, Faecalibacterium prausnitzii, Lactobacillus* may reduce the growth of CRC^4^. However, the impacts of many probiotics in cancer development remain to be characterized. Defining regulations of these effects and the underlying regulatory molecular mechanisms can facilitate therapeutic efficacy in CRC, and provide the potential of various treatment applications.

*Clostridium butyricum (C. butyricum or* C.B), a Gram-positive bacterium, consumes dietary fibers through fermentation to generate short-chain fatty acids (SCFAs), such as butyrate and acetate. Butyrate produced by C.B through the butyrate kinase pathway is the major fermentation product. C.B and metabolite butyrate have been safely used^10^ to treat disease such as diarrhea. However, mechanistic studies of the C.B-associated protective or ameliorative effects in cancer or diseases have not been fully characterized. Since roles of probiotic bacteria C.B in CRC development are not well characterized, we aim to identify the physiological contribution/molecular signals of anticancer effects from C.B in CRC such as cell proliferation, regulating apoptosis/metastasis, producing healthy metabolites, maintaining drug sensitivity of cancer, and improving immunotherapy efficacy.

Here, we show that C.B regulates many host processes, including cell proliferation, patient derived organoid (PDO) growth, metastases, immunotherapy, and chemotherapy responses. We have characterized the C.B-involved regulators during CRC growth, including p21, MYC, GADD45, Cyclin D, CDK4, and 1433 σ,^11,12^. Our results shed light on C.B-mediated MYC degradation, and illustrate a role of C.B in regulating Myc downstream target gene Thymidine synthesase (TYMS) through transcriptional regulation. In line with this observation, C.B overcomes the unresponsiveness issue of 5-FU in CRC treatment. In preclinical animal models, the host response to 5-FU cancer treatment and anti-PD1 immunotherapy was particularly improved by administrating C.B. Our study uncovers C.B as a potential anti-cancer agent regulating important aspects of drug resistance and immune surveillance escape during tumor treatment and provides insight into potential cancer treatment strategies.

## Results

### Clostridium butyricum is involved in regulating cell proliferation, patient derived organoid (PDO) growth, metastases in CRC

**Figure 1.**
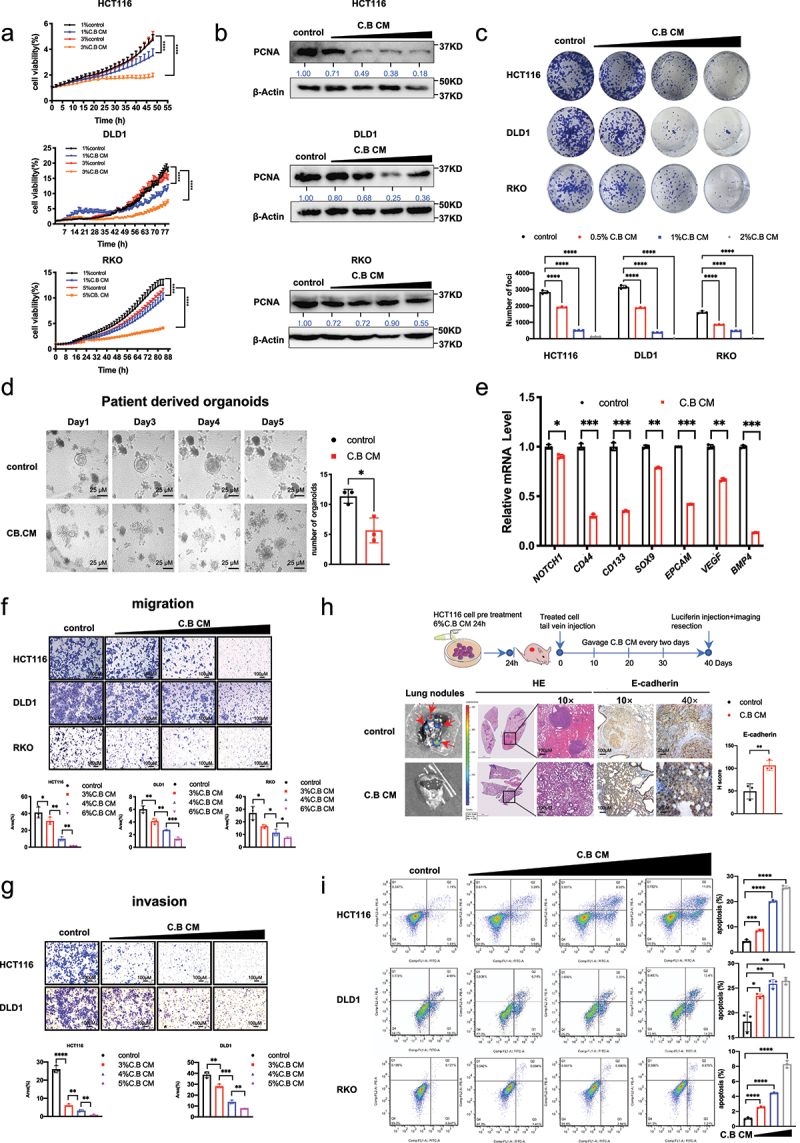
*Clostridium butyricum* regulates cell proliferation, migration and invasion, patient derived organoid (PDO) growth, and metastasis in CRC.

To investigate the potential antitumor effects of C.B, we prepared C.B conditioned medium (C.B CM) and treated CRC cells with different concentrations of C.B CM. The cell growth curves obtained by IncuCyte analysis and the colony formation assay indicated that C.B CM reduced the cell viability and proliferation of CRC cells when compared with control in a dose-dependent manner (Figures 1a, 1c). Importantly, the protein level of PCNA, a cell proliferation marker, was downregulated by C.B CM, which is consistent with the growth curves (Figure 1b). Meanwhile, C.B CM can also suppress proliferation of CRC patient-derived organoids (Figure 1d). We further check the several markers of stemness, such as NOTCH1, CD44, CD133, SOX9, EPCAM, VEGF, BMP4, and showed that C.B CM can downregulate the mRNA expression of those stemness markers (Figure 1e). Moreover, C.B CM can inhibit CRC cell migration and invasion compared to the control (Figures 1f,1g). To verify the results of cell experiments, we performed metastasis experiment employing the tail vein dissemination assay to analyze the C.B CM’s impact on the metastasis of CRC in animal model. C.B CM led to decreased number of lung metastasis (Figure 1h). Consistently, IHC staining demonstrated increased E-cadherin in C.B treated group (Figure 1h). Annexin V-FITC/PI staining was performed to determine the effect of C.B CM on apoptosis. Flow cytometry results showed that C.B CM treatment induced apoptosis in HCT116, DLD1 and RKO cells (Figure 1i). In summary, C.B CM inhibits colon cancer cell progression through regulating important cell functions.

(a) The growth curves of CRC cells treated with indicated concentrations of C.B CM (1%, 3% C.B CM for HCT116 and DLD1 cells, 1% and 5% C.B CM for RKO cells). Growth were measured by IncuCyte machine.

(b) Immunoblot analysis of PCNA protein expression in CRC cells (HCT116, DLD1 and RKO cells) treated with indicated concentrations of C.B CM. β-Actin was used as the internal control.

(c) Colony formation assay after treatment with indicated concentrations of C.B CM (0.5%, 1%, 2%) in CRC cells. The medium was changed every five days.

(d) Bright-field images and quantification of CRC patient-derived organoids after treatment with 1.5% culture medium or 1.5% C.B CM for 5 days. Scale bar: 25 μm.

(e) qRT-PCR analysis was performed to measure the mRNA levels of indicated stemness markers in HCT116 cells after treatment with or without 6%C.B CM.

(f) Transwell assays show the effects of treatment with indicated concentrations of C.B CM for 24 h CRC cells on the ability of cell migrations in CRC cells. Scale bar: 100 μm.

(g) Transwell assays show the effects of treatment with indicated concentrations of C.B CM on CRC cells for 24 h for the ability of cell invasions. Scale bar: 100 μm.

(h) Schematic overview of the intravenous metastatic dissemination assay via tail vein. HCT116-Luc tumor cells were treated with 6% C.B CM for 24 h before intravenous injection into BALB/c-nu mice (1 × 10^6^ cells per 200 µl per injection). IVIS imaging from lungs after luciferin injection on day 40 after tumor cell injection. HE and E-cadherin staining were performed. Scale bar: 10×, 100 μM; 40×, 25 μM.

(i) Flow cytometry analysis of apoptotic cells in CRC cells treated with different concentrations of C.B CM (3%, 4%, 5% C.B CM for HCT116 cells, 3%, 5% and 6% for DLD1 cells, 4%, 6% and 8% for RKO cells). Data are presented as mean ± SEM. ns, no significance; *, p < 0.05; **, p < 0.01; ***, p < 0.001; two-way ANOVA test, unpaired two-tailed Student test and one-way ANOVA test.

#### C.B is involved in regulating MYC ubiquitination and destabilization

**Figure 2.**
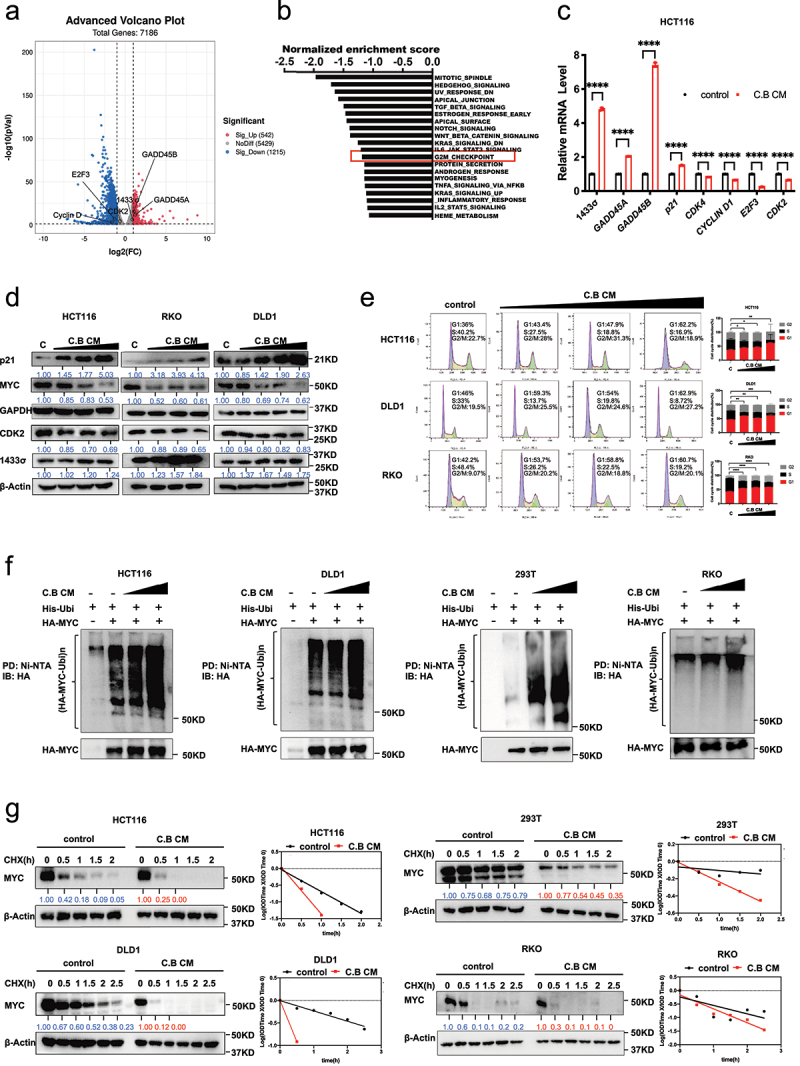
C.B instigates MYC ubiquitination and destabilization in regulating CRC progression.

The potential molecular mechanisms of C.B CM in inhibiting colon cancer growth were investigated by mRNA sequencing and analysis of differentially expressed genes (DEGs) in CRC. We treated HCT116 cells with C.B CM and profiled their gene expression. The volcano plot revealed that C.B CM changed the gene expression profile (Figure 2a). We further performed gene set enrichment analysis (GSEA), and the top 20 pathways significantly enriched with numerous DEGs in each biological process are listed. “HALLMARK_G2M_CHECKPOINT” pathway indicates cell proliferation regulation is involved (Figure 2b). As C.B CM can induce cell cycle arrest, we then check the expression of several genes related to cell cycle by real-time PCR. The result showed that C.B CM can influence the expression of genes associated with cell cycle such as p21, GADD45, Cyclin D, CDK4, 1433σ, and others (Figure 2c). Further, immunoblot analysis showed that C.B CM treatment can decrease the protein expression of MYC and CDK2, and increase the expression of cell cycle inhibitors, including p21 and 1433σ (Figure 2d). MYC expression level was particularly examined as high MYC expression can lead to proliferation, EMT, stemness, migration, invasion. We also analyzed cell cycle distribution by flow cytometry, and showed that C.B CM treatment caused significant G1 cell cycle arrest in HCT116, DLD1 and RKO cells when compared with no treatment control (Figure 2e). As MYC is usually regulated by ubiquitination, we then checked if C.B CM-mediated Myc downregulation involves ubiquitination machinery. Indeed, C.B CM can promote MYC ubiquitination and accelerate the turnover rate of MYC in CRC cells (Figure 2f-g). These data showed that C.B CM increased ubiquitin-mediated degradation of MYC, thereby decreasing MYC protein stability. Therefore, C.B CM may inhibit functions involved in CRC progression through reducing the stability of MYC.

(a) The volcano plot of differential gene expression in control and C.B CM-treated HCT116 cells (|logFC| > 0.5 and *p*-value<0.05).

(b) Representative top 20 pathways enriched in control group compared to C.B CM group based on GSEA analysis results.

(c) qRT-PCR analysis was performed to measure the mRNA levels of indicated cell cycle related genes after treatment with or without 6%C.B CM.

(d) Immunoblot analysis show the protein expressions of indicated cell cycle related proteins in CRC cells treated with increasing concentration of C.B CM (3%, 4% and 6% for HCT116, 3%, 4%, 5%, 6% for DLD1 cells, 4%, 5% and 6% for RKO).

(e) Flow cytometry analysis of cell cycle distribution of CRC cells upon C.B CM treatment. Cell cycle distribution in cells treated with different concentrations of C.B CM (3%, 4%, 5% for HCT116 cells, 4%, 5% and 6% for DLD1 cells, 3%, 4% and 6% for RKO cells) for 24 h was analyzed.

(f) Immunoblot analysis of ubiquitinated MYC protein in indicated cells treated with increasing concentration of C.B CM (3% and 6%) for 24 h. RKO was treated with C.B CM (4% and 8%). Cells were co-transfected with His-Ubi and HA-MYC plasmids. MG132 was added to the cells 6 h before they were harvested. The cell lysates were pulled down with Ni-NTA beads and immunoblotted with anti-HA antibody.

(g) The turnover rate of the c-Myc protein in indicated cells after treatment with 3%C.B CM for 24 h. RKO was treated with 4% C.B CM. CHX: cycloheximide, IOD: integrated optical density. Data are presented as mean ± SEM. ns, no significance; *, p < 0.05; **, p < 0.01; ***, p < 0.001; unpaired two-tailed Student test and two-way ANOVA test.

#### C.B antagonizes MYC-mediated expression of TYMS, thus sensitizing 5-FU treatment efficacy

**Figure 3.**
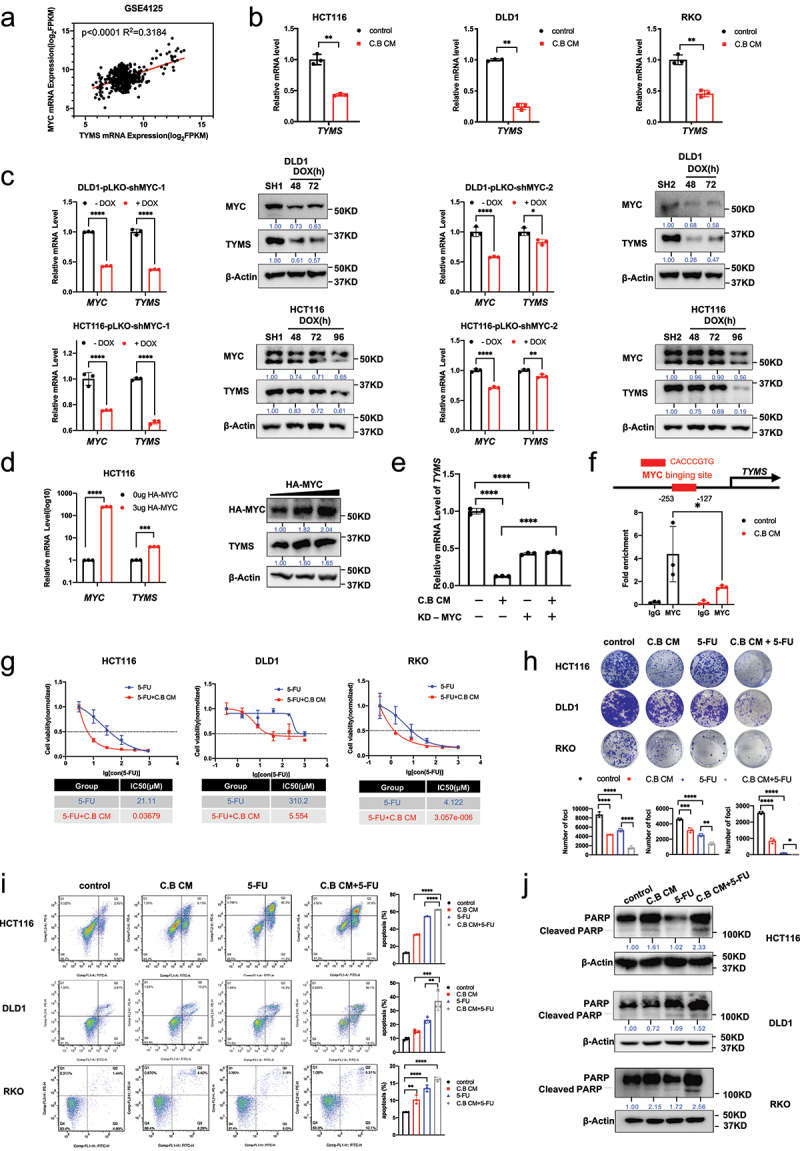
C.B attenuates MYC-mediated expression of TYMS to enhances the chemo sensitivity of CRC to 5-FU.

Thymidylate synthase (TYMS) is the important target for 5-fluorouracil (5-FU) chemotherapy^13^. 5-FU has been used for treating CRC cancer, but 5-FU drug resistance occurs, at least in part, due to upregulated TYMS, thereby leading to the prevalent unresponsiveness in patients. TCGA analysis shows that MYC is positively correlated with TYMS in terms of mRNA expression (Figure 3a), suggesting that targeting MYC to reduce TYMS may improve 5-FU sensitivity. We then hypothesized that C.B CM can improve the 5-FU chemo sensitivity in colon cancer cells by downregulating TYMS through the inhibition of MYC. We showed that C.B CM can downregulate mRNA level of TYMS in CRC cell lines (Figure 3b). To confirm TYMS was regulated by MYC in CRC cells, we performed MYC knockdown (KD) in HCT116 and DLD1 cells, and found that TYMS was downregulated after dox-induce knockdown MYC (Figure 3c). Consistently, MYC overexpression led to the upregulation of TYMS mRNA (Figure 3d). To investigate whether C.B regulates the expression of TYMS through its activity in degrading MYC, we performed MYC KD experiment. As expected, C.B and MYC KD can reduce the expression of TYMS; however, C.B-mediated downregulation of TYMS can be reversed by MYC KD to certain extent, suggesting that C.B-mediated downregulation of TYMS, at least in part, is regulated through its negative impact on MYC stability (Figure 3e).

(a) GSE4125 data base analysis showed that thymidylate synthase (TYMS) is positively correlated with MYC mRNA expression in CRC patients.

(b) qRT-PCR analysis was performed to measure the mRNA levels of TYMS in CRC cells after treatment with 6% C.B CM for 24 h.

(c) qRT-PCR and immunoblot analysis results showed changes of mRNA and protein levels of TYMS in pLKO-Tet-On-shMYC#1 and #2 expressing DLD1 and HCT116 cells after treatment with Doxycycline for several hours (48 and 72 hours for DLD1 cells, 48, 72 and 96 hours for HCT116 cells).

(d) mRNA and protein levels of TYMS in HCT116 cells transfected with the MYC expression plasmid.

(e) qRT-PCR analysis of TYMS in DLD1 cells treated under indicated situations. MYC induced KD with Doxycycline for 72 hours. Cells were treated with 3% C.B CM for 24 h.

(f) DLD1 cells were transfected with MYC expression plasmid, then treated with 6%C.B CM for 24 hours. The binding of MYC on TYMS promoter region were detected by ChIP-PCR in HCT116 cells.

(g) CCK8 assays were performed to detect IC50 of 5-fluorouracil (5-FU) when treated with or without 3%C.B CM in HCT116, DLD1 and RKO cells.

(h) Colony formation assay were performed to show the synergistic effect of 3%C.B CM and 5-FU (3 μM for DLD1 and RKO cells, 2.8 μM for HCT116 cells) in CRC cells. The cells were plated in 12-well plates for 9 days, and the medium was changed every five days.

(i) Flow cytometry analysis of apoptosis in CRC cells treated with 3%C.B CM and 5-FU treatments for 24 h. (DLD1 cell: 200 μM 5-FU, HCT116 cell: 100 μM 5-FU, RKO cells: 150 μM 5-FU).

(j) The protein level of cleaved PARP in 3% C.B CM, 5-FU (250 μM) and 3% C.B CM combination with 5-FU (250 μM) in HCT116, DLD1, RKO cells. Data are presented as mean ± SEM. ns, no significance; *, p < 0.05; **, p < 0.01; ***, p < 0.001; one-way ANOVA test, unpaired two-tailed Student test, and two-way ANOVA test.

ChIP-qPCR further demonstrated that MYC binds to the promoter regions (−253~-127) of TYMS. Importantly, MYC’s binding to this promoter region was attenuated after C.B CM treatment (Figure 3f). Based on the results above, we hypothesized that C.B CM may sensitize 5-FU’s drug effect on CRC. As shown in the analysis of CCK8 assay, in comparison to 5-FU alone, the combination of 5-FU and C.B CM showed lower IC50 (Figure 3g), suggesting that C.B CM can sensitize 5-FU’s drug effect. Congruently, the combination of 5-FU and C.B CM reduced the capacity for foci formation more efficiently than 5-FU or C.B CM alone (Figure 3h). Similar results were also observed in the apoptosis assay. Combination treatment with C.B CM and 5-FU notably increased the percentage of apoptotic cells based on Annexin V staining (Figure 3i). Further, the cleaved PARP protein expression is increased when compare with C.B CM or 5-FU alone (Figure 3j). Collectively, these results indicate that C.B CM sensitizes 5-FU treatment efficacy.

#### C.B potentiates 5-FU treatment in xenograft mouse model

**Figure 4.**
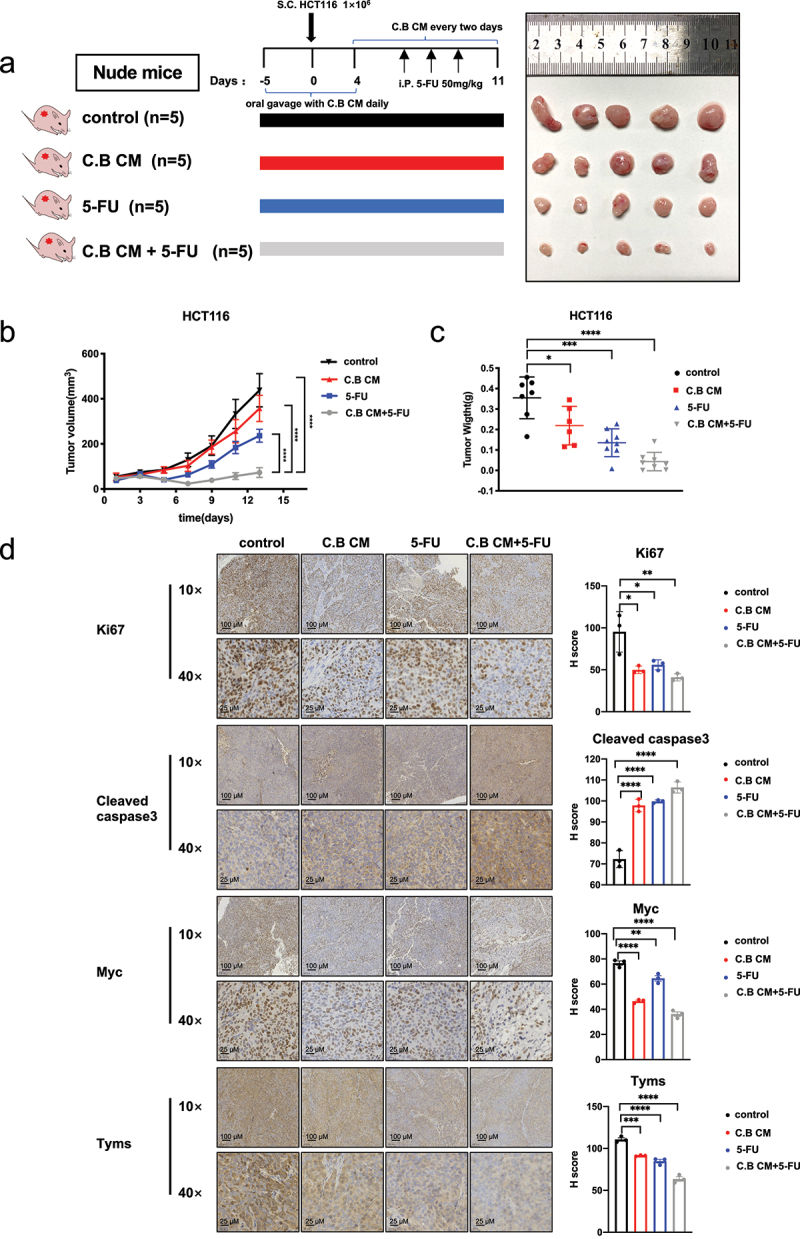
C.B boosts 5-FU treatment efficacy in mouse xenograft CRC model.

To confirm the synergistic effect of C.B CM plus 5-FU in suppressing CRC growth in vivo, we performed subcutaneous xenograft model in nude mice. Before inoculating HCT116 cells (1 × 10^6^ to the mice, we used oral gavage to administer C.B CM or culture medium (as control) daily until the tumor volume reach to 10 mm^3^. The schematic overview of in vivo experiment and representative images of all groups were demonstrated (Figure 4a). The volume and weight of tumors were decreased in the groups that received C.B CM, 5-FU, or both C.B CM and 5-FU when compared with control group. Importantly, the therapeutic efficacy of 5-FU was improved by C.B CM as the combination treatment inhibited the tumor growth much more effectively. (Figure 4b,c). IHC assays showed that C.B CM + 5-FU group have lower expression level of Ki67 and higher expression level of CASPASE 3, lower MYC and TYMS when compared with 5-FU alone (Figure 4d), indicating that C.B’s negative impact on MYC-TYMS axis can be recapitulated in mouse xenograft CRC model. Our data indicate that C.B CM can improve the therapeutic efficacy of 5-FU chemotherapy in CRC through interfering MYC-TYMS regulation.

(a) Schematic overview of in vivo experiment and representative images of HCT116 cells xenograft tumor in control (n = 5), C.B CM (n = 5), 5-FU (n = 5), and C.B CM + 5-FU (n = 5) groups.

(b) Tumor volumes of indicated groups.

(c) Tumor weights of indicated groups.

(d) Represent IHC images and quantification analysis of Ki67, CASPASE 3, TYMS and MYC from indicated groups. Staining intensity was quantitated. Scale bar: 10×, 100 μM; 40×, 25 μM. Data are presented as mean ± SEM. ns, no significance; *, p < 0.05; **, p < 0.01; ***, p < 0.001; one-way ANOVA test and two-way ANOVA test.

### Therapeutic benefits of C.B involve Myc downregulation, increased CD8+ T cell infiltration, thereby boosting anti-PD1 therapeutic efficacy

**Figure 5.**
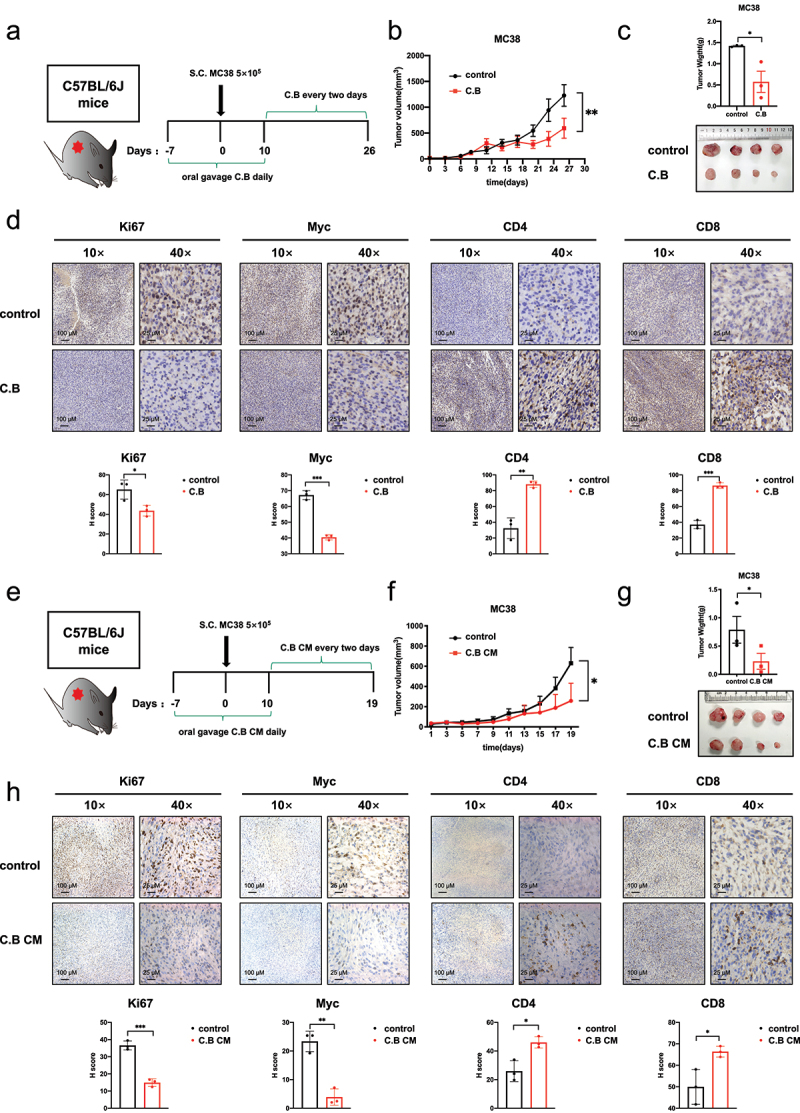
Therapeutic benefits of C.B by downregulating MYC expression and enhancing the immune response through increased CD8+ cell infiltration.

Various evidence has shown that C.B can modulate the immune reactants to confer the protective effect to ameliorate disease conditions. To investigate further whether the signal regulators we have identified from C.B, such as MYC, are involved in shaping the regulation of the immune reactants, we establish a syngeneic mouse CRC model. Immune-competent C57BL/6J mice were engrafted with mouse MC38 colon adenocarcinoma subcutaneously and were administered with C.B (Figure 5a). Live bacteria of C.B were used as we can assay the host-microbe interaction and assess this impact on tumor growth. C.B has an inhibitory effect on the growth of subcutaneous tumor (Figure 5b-c). Meanwhile, the IHC staining showed that C.B treatment decreased the expression of Ki67 and MYC, while the expression of CD4 and CD8 markers of T cell infiltration were increased (Figure 5d). C.B CM contains the metabolites or active molecules that can directly impact on host functions. Similarly, the tumor growth of MC38 model treated with C.B CM was analyzed (Figure 5e). As shown in treatment with C.B CM, the tumor volumes and weights were decreased compared with the control group (Figure 5f-g). The IHC staining shows that C.B CM treatment not only decreased the expression of Ki67 and MYC but also increased the expression of CD4 and CD8 markers (Figure 5h). Collectively, these results demonstrate that both C.B and C.B CM exert an antitumor effect in CRC and imply that both anti-cancer effects of C.B and C.B CM involve MYC downregulation and tumor immunity/T cell infiltration.

(a) Schematic diagram of the C57BL/6J mice model and C.B treatment schedule.

(b) Tumor growth curves of control (n = 4) and C.B groups (n = 4) were shown. Statistical differences between untreated and the treated animals were analyzed by applying the two-way ANOVA test.

(c) Tumor weights and representative IHC images of tumors in control and C.B groups were shown.

(d) Represent images of Ki67, Myc, CD4 and CD8 immunohistochemistry staining in tumor tissue from control and C.B groups. Staining intensity was quantitated. Scale bar: 10×, 100 μm; 40×, 25 μm.

(e) Schematic diagram of the C57BL/6J mice model and C.B CM treatment schedule.

(f) Tumor growth curves of control (n = 4) and C.B CM groups (n = 4) were shown. Statistical differences between untreated and the treated animal were analyzed by applying the two-way ANOVA test.

(g) Tumor weights and representative images of tumors in control and C.B CM groups were shown.

(e) Represent images of Ki67, Myc, CD4 and CD8 immunohistochemistry staining in tumor tissue from control and C.B CM groups. Staining intensity was quantitated. Scale bar: 10×, 100 μm; 40×, 25 μm. Unpaired two-tailed Student test, two-way ANOVA test.

**Figure 6.**
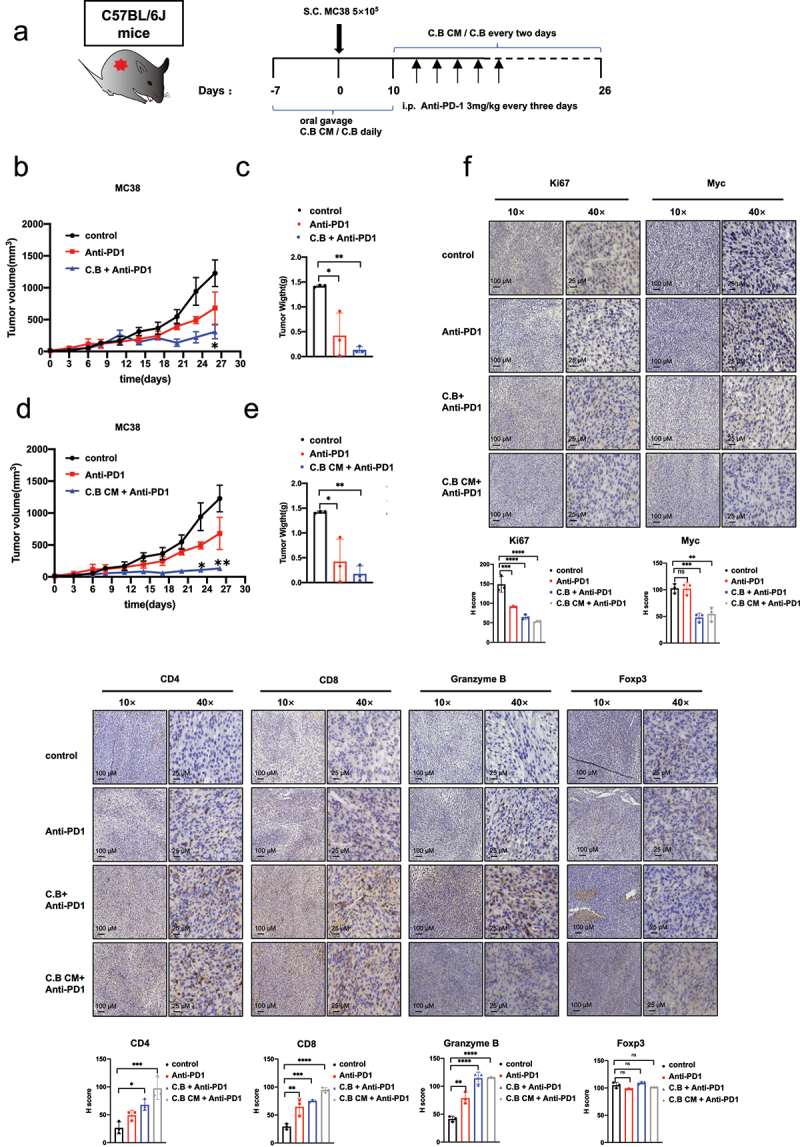
C.B or C.B conditioned medium significantly boost the efficacy of anti-PD1.

Immunotherapy with immune-checkpoint inhibitors (ICIs) has revolutionized the treatment of various types of cancer. However, the majority of patients, including CRC, receiving these ICI therapies still have limited clinical benefit. There is an urgent need to boost the efficacy of ICI immune therapy. To further explore whether C.B and C.B CM can enhance the immunotherapeutic efficacy of anti-PD-1 in colorectal cancer, we established again MC38 tumor model in immune-competent C57BL/6J mice treated with C.B and anti-PD1 (Figure 6a). The volume and weight of tumors were both decreased in the anti-PD1 and C.B+anti-PD1 groups when compared with control group. Importantly combination treatment (C.B+anti-PD1) showed better efficacy than anti-PD1 treatment group, suggesting that C.B can boost the therapeutic efficacy of anti-PD1 (Figure 6b-c). Moreover, C.B CM has the same efficacy as C.B in enhancing anti-PD1 immunotherapy (Figure 6d-e). The IHC staining shows that combination treatment increases the expression of CD4, CD8 and Granzyme B more efficiently than anti-PD1 alone (Figure 6f). Together, C.B or C.B conditioned medium significantly boost the efficacy of anti-PD1 by shaping the immune-landscape of the tumor environment/tumor immunity, thereby enhancing antitumor therapy of anti-PD1 to increase the tumor survival.

(a) Schematic diagram of the C57BL/6J mice model and treatment schedule.

(b) Tumor growth curves of control (n = 4), anti-PD1 (n = 4), and C.B + anti-PD1 groups (n = 4) were shown.

(c) Tumor weights of tumors in control, anti-PD1, and C.B + anti-PD1 groups were shown.

(d) Tumor growth curves of control (n = 4), anti-PD1 (n = 4), and C.B CM + anti-PD1 groups (n = 4) were shown.

(e) Tumor weights of tumors in control, anti-PD1, and C.B CM + anti-PD1 groups were shown.

(f) Represent images of Ki67, Myc, CD4, CD8, Granzyme B, and Foxp3 immunohistochemistry staining in tumor tissue from control and C.B groups. Staining intensity was quantitated. Scale bar: 10×, 100 μm; 40×, 25 μm. Data are presented as mean ± SEM. ns, no significance; *, p < 0.05; **, p < 0.01; ***, p < 0.001; unpaired two-tailed Student test, two-way ANOVA test and one-way ANOVA test.

## Discussion

**Figure 7.**
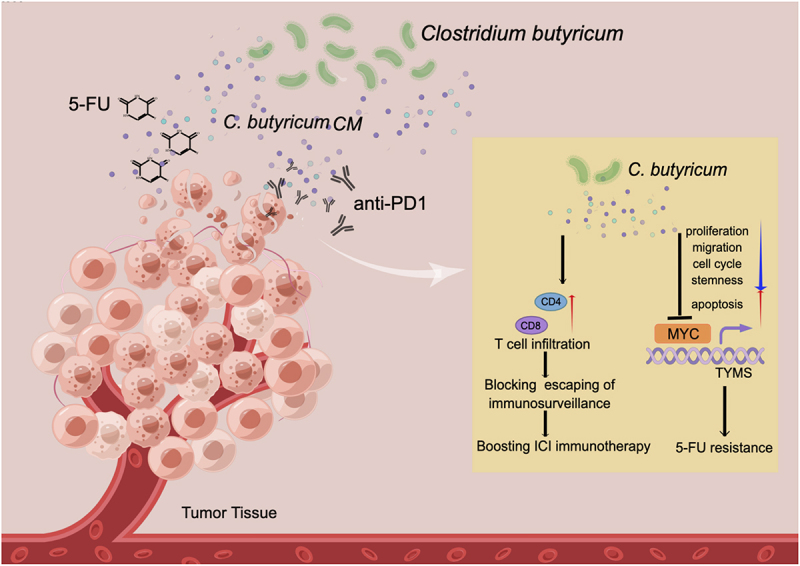
Schematic summary of C.B’s role in modulating 5-FU drug resistance and boosting anti-PD1 immunotherapy.

Various studies have explored associations between microbiota and host physiological functions; however, characterizing the molecular mechanisms behind these associations is quite challenging. C.B is a butyrate-producing bacterium that functions not only as a probiotic but also demonstrates potential protective or ameliorative effects in terms of tumor treatment. It actually links its activity to boost immunotherapy efficacy in certain cancer, but our knowledge about these impacts and targets of C.B has not been well characterized. Here we characterize that C.B is a critical negative regulator of MYC signaling pathways: involved in growth inhibition, drug sensitization, and boosting immunotherapeutic therapy efficacy. Our data shed light on C.B’s signaling targets and reveal how MYC, a major target of C.B, is intercepted by C.B to ameliorate tumorigenesis (Figure 7).

C.B has a negative impact on MYC stability, thereby reducing TYMS expression and subsequent attenuation of 5-FU resistance. C.B enhances CD4/CD8 T cell infiltration, thereby blocking escaping of immunosurveillance and boosting the efficacy of anti-PD1 (ICI) immunotherapy. The illustration was prepared by Figdraw.

MYC is one of the most commonly activated oncoproteins in human cancer^14^. MYC activation leads to manifestation of many hallmarks of cancer involved in cancer growth. In preclinical models, inactivation of MYC leads to sustained tumor regression, so this concept of inhibiting MYC has been pursued as a cancer targeting strategy^15^. MYC is one of the well-characterized transcription factors, including OCT4, SOX2, NANOG, KLF4, in cancer stem cell establishment, maintenance, and functions^16^. We showed that C.B is competent to suppress several stemness markers. It is possible that C.B-mediated MYC ubiquitination and degradation may, at least in part, account for C. B’s impact on suppressing PDO growth and reduced stemness. We also demonstrated that C.B can hinder mouse metastasis model. It is not clear how C.B can attenuate cancer metastasis although E-cadherin was increased by C.B. Because MYC is a driver of metastasis^17^, it is then conceivable that C.B-mediated MYC ubiquitination and degradation leads to attenuated metastasis. Also, it has been shown that intracellular or tumor-resident bacteria can travel with the tumor cells and promote metastatic colonization^18^. C.B may alter the composition of microbiota in tumor microenvironment, thereby mitigating tumor-resident bacteria’s impacts on metastasis. It remains to be determined behind C.B-mediated block of cancer metastasis.

Thymidylate synthase (TYMS), an enzyme has a central position in the pathway of DNA synthesis, is an important target for 5-fluorouracil (5-FU) chemotherapy^13^. 5-FU has been used for treating CRC cancer, but 5-FU has toxicity and usually causes the prevalent unresponsiveness in patients, resulting in limited clinical benefits. It is then urgent to identify a strategy to enhance the efficacy of 5-FU. It turns out that TYMS can be transcriptionally regulated by MYC. Given that C.B supplementation leads to the destabilization of MYC and that MYC – mediated TYMS upregulation may lead to unresponsiveness of 5FU therapy, we rationalize that C.B will overcome the unresponsiveness issue of 5-FU via MYC destabilization/TYMS reduction. In a resistance study of 5-FU, it was shown that sensitivity was related to levels of TYMS in chemotherapy^13^. Consistently, TYMS overexpression leads to clinical resistance to 5-FU-based chemotherapy^19^. Here we showed that C.B-mediated MYC degradation, thereby reducing TYMS expression, which in turn sensitizes the efficacy of 5-FU therapy. The link between C.B and the signaling pathway of MYC, a pivotal transcription factor involved in 5-FU drug resistance, is documented in our studies. Our results implied that C.B administration can be employed in clinical for overcoming 5-FU drug resistance. It is possible that C.B may be used for other type of drug resistance since microbiota has been shown to be involved in drug efficacy^20^.

Various studies have demonstrated that the gut microbiome can affect the response to immune checkpoint inhibitors (ICIs) in cancer patients,^21,22^. For example, in metastatic renal cell carcinoma, progression-free survival was significantly longer in cancer patients receiving nivolumab and ipilimumab with C.B (Strain MIYAIRI 588) than without, suggesting that C.B (Strain MIYAIRI 588) can benefit the clinical outcome^23^. However, the detailed mechanisms remained to be determined although the circulating cytokines, including ILI1β, IL10, IL12 and TNF α, have been changed. Further, C.B is also documented in regulating immunity in other disease. For example, *Clostridioides difficile* infection (CDI) causes life-threatening diarrhea and colitis. In animal model, C.B treatment significantly ameliorates clinical symptoms associated with CDI via increasing the number of neutrophils and Th1 and Th17 cells in mice^24^, suggesting that modulating the immune reactants is critical in conferring the protective effect of C.B.

Detailed studies remain to be performed to investigate C.B’s effects in boosting immunotherapy. However, uncharacterized metabolites from C.B could be immunogenic. *Akkermansia muciniphila* generates an immunogen, a lipid (diacyl phosphatidylethanolamine) isolated from *A. muciniphila’s* cell membrane, to induce inflammatory cytokines for affecting host immune signaling^25^. It is then possible that C.B’s metabolites may have immunoregulatory effects on host, thereby affecting immunotherapeutic efficacy. We have performed the metabolomics to determine the metabolites generated from C.B (Figure S1). For example, one of the major metabolites – butyrate is known to induce the differentiation of colonic Treg cells^26^. Further, we have performed some of the critical experiments using butyrate, and demonstrated that butyrate is effective in regulating important biological functions in cancers, including inhibiting cell growth, reducing cell migration, eliciting apoptosis, causing cell cycle arrest, downregulating Myc, enhancing Myc turnover in CRC cell line (Figure S2). Thus, butyrate’s role in multiple biological functions may affect immunotherapy. Studies on more C.B metabolites, which we have identified (Figure S3), involved in inhibiting cell growth or other functions warrant further investigation.

We demonstrated that C.B can obviously boost the response in immunotherapy of CRC by increasing CD8/CD4 T cell infiltration. Thus, synergy between immunotherapy and C.B supplementation leads to augmenting the efficacy of ICIs in preclinical model. This observation is particularly important as numerous patients respond poorly to ICI^27^, including CRC. Unresponsiveness to ICIs remains a significant challenge to highly expected immunotherapeutic strategies. Thus, there is an urgent clinical need to design effective therapeutic approaches for mitigating ICI unresponsiveness in patients. We showed that C.B administration pose an application potential for ICI-treated CRC. Further, the expression of PD-L1 in tumor is upregulated by MYC^28^, which causes escape of immunosurveillance, leading to unresponsiveness or resistance to ICI. We showed that C.B has reduced the stability of MYC, which may account for, at least in part, its boosting impact on ICI treatment efficacy.

In summary, our data uncover a network of C.B signaling: MYC stability regulation, metastases, drug resistance, immunosurveillance, and tumorigenicity. The role of C.B in destabilizing MYC stability through enhanced ubiquitination highlights important mechanisms of C.B’s impact on regulating 5-FU sensitization and increasing therapeutic potential of ICIs in CRC. Harnessing these anti-cancer effects of C.B could facilitate the development of more therapeutic designs to manage CRC.

## Materials and methods

### Cell culture

The human DLD1, HCT116 and RKO colon cancer cell lines and HEK293T were purchased from the American Type Culture Collection (ATCC). All cells were maintained at 37°C and 5% CO_2_. HEK293T and HCT116 cells were grown in Dulbecco’s modified Eagle’s medium (DMEM), while DLD1 cells were maintained in RPMI/1640, and RKO cells were maintained in Minimum Essential Medium (MEM). All mediums were supplemented with 10% FBS and 1% penicillin – streptomycin. The medium was changed every other day, and cells were passaged using 0.25% trypsin/EDTA. All experiments were performed using the cells within 3–10 passages.

### Conditioned medium and Bacteria preparation

*C. butyricum* (ATCC 19,398) was supplied by the China General Microbiological Culture Collection Center. *C. butyricum* was inoculated into 100 ml cooked meat medium base (culture medium) under anaerobic conditions for 24 h at 37°C. C.B was collected when OD reached 0.8. After centrifugation (3000 g × 5 min) the supernatant was filtered through Millex-GP Filter Unit (0.22 μm pore size, Millipore), and then stored at −80°C. This is the C. B conditioned medium (C. B CM), which can be further diluted with complete cell culture medium for use.

In the cell experiment, we used the volume ratio method to conduct the relevant treatments. For animal models, we employed C.B CM 200 μl to perform the experiments. 200 μl of C.B CM is administrated and is equal to approximately 10 mg/kg. C.B is a living bacterium, and we used 1 × 10^8^ CFU to treat animal.

### Reagents and Antibodies

5-Fluorouracil (Aladdin， #F100149–25 g), DMSO (MP Biomedicals， #Q6949)，Anti-mouse PD-1 (CD279) (BioXcell, #BE0146-50 MG), Hydroxyphenyllactic acid (MCE, #HY-113219), Sodium butyrate (aladdin, #S102956), Sodium Acetate (1,2-13C2, 99%) (CIL, #CLM-440-1), Alpha-Ketoglutaric acid (MCE, #HY-W013636), Fumaric acid (MedChemExpress(MCE, #HY-W015883), 2-Ketoglutaric acid (MCE, # HY-W013636), Nicotinic acid (MCE, #HY-B0143), Antibodies against Flag-Tag (Cell Signaling, #8146), HA-Tag (Cell Signaling, #3724), anti-GAPDH (Proteintech #60004–1). PARP (Cell Signaling, #532S)，Anti-PCNA(Abcam, #ab29), P21 (Cell Signaling, #2947S), c-Myc (Cell Signaling, #13987S), Cleaved Caspase-3 (Cell Signaling, #9664P), Anti-14-3-3 (Abcam, #ab14123), CDK2 (Cell Signaling, #18048), β-Actin (Sigma-aldrich #A5441-2 mL), TYMS (Proteintech, #15047–1-AP), Ki67 (Proteintech, # 27309–1-AP), CD4 (Cell Signaling, # 25229S), CD8 (Cell Signaling, # 98941s), Granzyme B (D6E9W) (Cell Signaling, #46890), Foxp3 (Cell Signaling, #12653), E-cadherin (Cell Signaling, # 93195s).

### Cell Proliferation Assay and IC50 calculation

For growth curve, cell viability was monitored by the IncuCyte live cell analysis system (Essen BioScience, Ann Arbor, MI, USA). To assess the cell proliferation, CRC cells were equally seeded into fresh 12-well plates at a density of 1 × 10^5^ cells. Cells were treated with different indicated concentrations of C.B CM. After placing the cell in a cell incubator, the IncuCyte live cell analysis system analyze the photographs at 9 random fields to obtain growth curve.

For IC50 calculation, cell viability was determined by Cell Counting Kit-8 (K1080, Apexbio Technology LLC, Houston, TX, USA). CRC cells were seeded into 96-well plates for 24 h. The cells were treated with indicated doses of C.B CM, 5-FU, and the combination of C.B CM and 5-FU for 48–72 h. Following that, 10% (v/v) CCK-8 solution was prepared and added to every well, and cells were plated into a cell incubator for 24 h. Optical density was measured at 450 nm using Epoch™ Multi-Volume Spectrophotometer and Take3™ (BioTek, Winooski, VT, USA). Cell viability was normalized as a percentage of the negative controls treated with culture medium. IC50 values were calculated by using linear regression equations obtained from the concentration vs percentage of viable cells.

### Colony formation

The HCT116, DLD1 and RKO cells were seeded into 12-well plates (BD Falcon, Franklin Lakes, NJ, USA) at a density of 500 cells per well. To determine the antitumor effect of C.B CM, cells were treated with different indicated concentration of C.B CM after 1 days of adherent culture, with 9 days of maintenance in DMEM medium (HCT116), RPMI medium 1640 (DLD1 cell) or MEM medium (RKO cell) supplemented with 10% FBS and 1% penicillin and streptomycin. To determine the synergistic antitumor effect of 5-FU and C.B CM, after 1 days of adherent culture, cells were treated with 5-FU (HCT116, 2.8 μM; DLD-1, 3 μM; RKO 2.5 μM), 3% C.B CM, or both 5-FU and 3% C.B CM for 9 days. After washing three times with PBS, the colonies were fixed with 4% polyoxymethylene (15 min) and stained with 0.05% crystal violet (10 min) at room temperature. Number of colonies was calculated by Image J (NationalInstitutes of Health, Bethesda, MD, USA).

### Immunoblotting

Briefly, cells were lysed in lysis buffer (Tris（50 mM）, NaCl (150 mM), 0.1% NP-40 and 1% Triton X-100) with protease inhibitors plus vortexing or sonication at 4°C. Lysate samples were loaded and run on SDS-PAGE gels, then transferred onto PVDF membranes (Merck millipore, #IPVH0001). For immunoblotting, protein-transferred membranes were blocked with TBST buffer (10 mM Tris-HCl (pH 7.9), 150 mM NaCl, and 0.05% Tween 20) with 5% skim milk, incubated with primary antibodies overnight, subsequently incubated with HRP-conjugated secondary antibodies 1 hour, and detected by ECL western blotting detection reagents (GE Healthcare, RPN2209).

### Organoid culture

Human fresh CRC organoid culture was performed as previously described^29^. CRC patients’ tumor tissue (moderately differentiated adenocarcinoma of rectum, Grade II) is obtained from The Sixth Affiliated Hospital of Sun Yat-sen University under approved IRB. Tumor tissue was washed with cold phosphate-buffered saline (PBS) and cut into small pieces, then digested with EDTA. After digesting into clumps of cells, the sample was seeded into Matrigel in 24-well plates. Following Matrigel polymerization (10 min at 37°C), 500 μl human culture media (Advanced DMEM/F12 containing, 1× N2 (Life Technologies), 10 mM HEPES, 2 mM GlutaMAX, 1× B27, 10 nM gastrin I (Biogems), 50 ng/ml recombinant EGF, 500 nM A83–01 (Biogems), 100 ng/ml recombinant Noggin (Peprotech), 500 ng/ml R-spondin- 1 (Peprotech), 10 μM SB202190 (Sigma) 10 μM Y-27632 (Abmole), 10 mM nicotinamide (Sigma), 1 mM N-acetylcysteine (Sigma) and penicillin/streptomycin. The sample was treated with 1.5% culture medium and 1.5% C.B CM, then was changed with the fresh culture medium every 3 days. The organoid numbers in each well were quantified at day 5.

### Real-time quantitative PCR (qPCR)

Total RNA was extracted with TRIzol (Invitrogen) and reverse transcribed using ReverTra Ace® qPCR RT Master Mix with gDNA Remover (TOYOBO). Real-time PCR was performed using the 2× SYBR Green qPCR Master Mix (bimake) in a LightCycler® 480 II (Roche) instrument. The qPCR primers are listed in Table S1.

### Migration and invasion assay

For the migration and invasion assay, 8.0 µm PET track-etched membrane transwell (BD Falcon, Franklin Lakes, NJ, USA), with each transwell inserted into the 24-well plates (NEST, Shanghai, China) to form an upper and a lower chamber, and Matrigel (BD) were used for estimating cell invasion. HCT116, DLD1 and RKO cells were pretreated with different indicated concentration of C.B CM for 24 h. Then C.B CM treated cells (1 × 10^5^ cells for migration, or 1.5 × 10^5^ cells for invasion) in 200 µL of serum-free media were seeded into upper chambers. RPMI 1640 or DMEM supplemented with 10% FBS was placed in the lower chamber. Migration and invasion were scored at 12 and 24 h, respectively. The migrated cells in the lower layer of the chamber were fixed with 4% paraformaldehyde for 5 min at room temperature, and stained with crystal violet for 15 min. Photographs were taken for counting under a microscope.

### Metastasis mice model

Male BALB/c nude mice (5-to-6-week-old, 16–20 g) were intravenously injected with 1 × 10^6^ per 200 μl of HCT116-Luc cells via the tail vein and performed as previously described^30^. Briefly, cells were pretreated with 6% C.B CM or 6% culture medium for 24 h. Then oral gavage was carried out with 200 μl C.B CM or culture medium in mice every two days. After 40 days, mice were intraperitoneally injected with VivoGlo Luciferin （promega，#P1043, 100 μl per 10 g mouse weight). 10 min later, signals were detected using the In Vivo Imaging System (IVIS). Macroscopic counting of metastasis nodes of the lungs was performed.

### Flow Cytometry

Flow cytometric analysis of cell apoptosis was detected by using the Annexin V-FITC/Propidium Iodide (PI) Apoptosis Detection Kit (LiankeBio, Hangzhou, China). CRC cells were plated in 6-well plates in complete medium. 24 h later, different indicated concentration of C.B CM or 5-FU were dissolved into fresh medium to replace the old medium. Cells were treated for 24 h, and then both supernatant and adherent cells were collected by trypsinization. The cell suspension used for analysis was prepared with 500 μL cold PBS containing around 1 × 10^6^ cells. Annexin V-FITC (10 μL) and PI (5 μL) were added, respectively, and stained at room temperature in the dark for 5 min. The pretreated cells were analyzed by flow cytometry (CytoFLEX, Beckman Coulter, Inc., Miami, FL, USA).

For cell cycle distribution analysis, a propidium iodide (PI) cell cycle staining kit (LiankeBio, Hangzhou, China) was used according to the manufacturer’s instructions. The cell cycle phases were determined by flow cytometry (CytoFLEX, Beckman Coulter, Inc., Miami, FL, USA).

### RNA sequencing

RNA-seq was carried out on the BGISEQ-500 platform using the single-end 50 bp protocol as previously described^31^. HCT116 cells were grown with culture medium or C.B CM. Total RNA was harvested from HCT116 cells after treatment. The mRNA with poly A tail was enriched by magnetic beads with Oligod T. The RNA obtained was segmented by interrupting buffer, the random N6 primers were used for reverse transcription, and then the cDNA two-strand was synthesized to form double-stranded DNA. The end of the synthesized double-stranded DNA is blunted, and the 5’ end is phosphorylated, while the 3’ end forms a sticky end with an “A” protruding, and then a bubbling linker with a protruding “T” at the 3’ end is connected. The constructed cDNA library was inspected and sequenced after being qualified. The ligated products are amplified by PCR with specific primers. The PCR product was heat-denatured into single-stranded DNA, and then a single-stranded circular DNA library was obtained by circularizing the single-stranded DNA with a bridge primer. The constructed library is qualified by a standard cDNA library and sequenced after it is qualified. Subsequently, sequenced data filtering with quality score was performed on sequenced raw reads. GSEA software (Broad Institute, Cambridge, MA, USA) was used to operate gene set enrichment analysis and to search for the biological change between groups.

### Transfection, Lentivirus generation, and infection

The human *MYC* gene was subcloned into pCDNA3.1 to generate constructs with HA-tag or FLAG-tag. Cells were plated in 6-well plate at a density of 2 × 10^5^ cells/well. 24 h later, cells were transfected with the indicated plasmids using polyethylenimine HCl MAX, Linear, Mw 40,000 (PEI MAX 40,000) (polysciences Inc) according to the manufacturer’s instructions. To prepare lentivirus for the knockdown of MYC, MYC short hairpin RNA (shRNA) was generated with the shMYC–1:GATTTGGAAGAGGCGAGATAA and shMYC–2: CCCAAGCGACTCGGGTAAGGA oligonucleotides targeting the MYC transcript. These shRNAs were ligated into the pLKO-Tet-On vector. To produce lentiviral particles, 1 × 10^7^ HEK293T cells in a 55-cm^2^ dish were co-transfected with mixture of 10 μg MYC shRNA construct, 5 μg of psPAX2, 5 μg pMD2.G and 25 μl PEI. The supernatant containing viral particles was harvested at 48 and 72 h after transfection, and filtered through Millex-GP Filter Unit (0.45 μm pore size, Millipore). To infect cancer cells with lentivirus, cells were seeded respectively in 6-well plates and infected twice with cell culture medium containing 2 mL lentivirus, 200 μL FBS and 5 mg/mL polybrene (Sigma) at 37°C for 48 and 72 h. To increase the knockdown efficiency, infected cells were selected with puromycin (2.5 ~ 5 μg/mL) for 48 h.

### Turnover assay

Protein turnover was performed as previously described^32^. Briefly, HCT116, DLD1 and HEK293T cells were treated with 3% culture medium or 3% C.B CM, RKO cell was treated with 4%. 24 h later, cells were treated with 60 μg/ml cycloheximide and harvested at 0, 0.5, 1, 1.5, 2 or 2.5 h after cycloheximide treatment. The protein levels were analyzed by western blotting.

### Ubiquitination assay

Ubiquitination assay was performed as previously described^33^. Briefly, HCT116, DLD1, HEK293T and RKO cells were co-transfected with 2 μg HA-MYC and 4 μg His-ubiquitin plasmids, and then treated with culture medium and increasing amount of C.B CM (3%, 6%, RKO was treated with 4% and 7%). Then all cells were treated with 50 μg/ml MG132 for 6 h. Cell lysates were pulled down with anti-HA or Ni-NTA agarose. The beads were boiled with 2× loading buffer for 5 min, after extensive washing. The protein levels were analyzed by western blotting with the indicated antibodies.

### ChIP assay

CHIP assay was performed as described^34^. After transfection with exogenous 12 μg HA-MYC, cells were treated with 6% culture medium or 6%C.B CM for 24 h, then collected to perform the ChIP assays, using EZ-ChIP Kits (Millipore) according to the protocol described by ^35^. Briefly, cells were treated as indicated treatment. After 48 ~ 72 h, cells were crosslinked for 10 min using 1% PFA and were then quenched with glycine. Cells were then lysed in cell lysis buffer to break the cell membrane. Then centrifuge the lysate and resuspended in nuclear lysis buffer and sonicated. After centrifugation, the supernatants were diluted as protocol required and incubated with 1 μg aliquots of primary antibodies (Anti-HA-tag, control rabbit IgG,) and ChIP beads overnight. Antibody bound protein/DNA complexes were then pulled down using ChIP beads and were washed with Low salt buffer, high salt buffer and LiCl buffer, TE buffer. After that, each ChIP samples were eluted with elution buffer. Eluted protein/DNA complexes were finally digested with protease K and purified DNA samples were analyzed by qRT-PCR for MYC binding sites.

### Xenograft CRC model

All animal studies were approved by the Animal Ethical and Welfare Committee of Sun Yat-sen University. To verify the synergistic effect of 5-FU and C.B CM, Male BALB/c nude mice (5-to-6-week-old, 16–20 g) were purchased from GemPharmatech (Nanjing, Jiangsu, China). HCT116 cells (1 × 10^6^ cells/mouse) xenograft mice were divided into four groups: the control group (*n* = 5), C.B CM group (*n* = 5), 5-FU group (*n* = 5), and C.B CM + 5-FU group (*n* = 5). Mice were carried out with gavage with C.B CM 200ul daily. Meanwhile, mice were treated with 5-FU (50 mg/kg, PBS) intraperitoneal (IP) injection 3 times after tumor inoculation. The control group was treated with the corresponding culture medium. The tumor length and width were measured every 3 days, and the mice were sacrificed after 16 days. The volume was calculated according to the formula (length × width2)/2.

To verify the synergistic effect of anti-PD-1 and C.B CM or C.B, male C57BL/6J mice were pretreated with C.B 1 × 10^8^ CFU or C.B CM 200ul daily before subcutaneously injection of MC38 cells (5 × 10^5 cells/mouse) at the flanks of the mice. The control group was treated with the corresponding culture medium. After several days, when tumor size reached 10 mm^3^, mice were treated with C.B or C.B CM oral gavage and intratumor injection every two days before sacrificed. Meanwhile, anti-PD-1 was treated every three days (i.p. 3 mg/kg, PBS) after tumor inoculation. The tumor length and width were measured every 3 days. The volume was calculated according to the formula (length × width2)/2.

### Immunohistochemistry (IHC)

Tumor tissues were fixed in 4% paraformaldehyde and were then embedded in paraffin by a company (Servicebio). Paraffin-embedded slides were deparaffinised in xylene and rehydrated in a graded ethanol series. Slides were processed for antigen retrieval by microwave heating for 15 min in 1× EDTA unmasking solution (Origene), cooled for 30 min and incubated in 3% hydrogen peroxide for 10 min. After blocking with blocking solution (Origene) for 1 h at room temperature, slides were incubated in diluted primary antibody overnight at 4°C. The next day, after incubation with biotinylated goat anti-rabbit or anti- mouse IgG at room temperature for 30 min, immunostaining was visualized with diaminobenzidine, and sections were then counterstained with hematoxylin. Antibodies against Ki67, CASPASE 3, MYC, TYMS, CD4, CD8, Granzyme B, Foxp3, E-cadherin were used.

### Metabolomic analysis of C. butyricum CM

An ultra-performance liquid chromatography coupled to tandem mass spectrometry (UPLC-MS/MS) system (ACQUITY UPLC-Xevo TQ-S, Waters Corp., Milford, MA, USA) was used to quantitate the metabolite of C. butyricum CM and culture medium. The raw data files generated by UPLC-MS/MS were processed using the TMBQ software (v1.0, Metabo-Profile, Shanghai, China) to perform peak integration, calibration, and quantitation for each metabolite. The self-developed platform iMAP (v1.0, Metabo-Profile, Shanghai, China) was used for statistical analyses, including PCA, OPLS-DA, univariate analysis and pathway analysis.

### Quantification and statistical analysis

Statistical analysis was carried out using the software GraphPad Prism 9.0 (GraphPad Software Inc., La Jolla, CA, USA). The volcano plot analysis was performed using the OmicStudio tools at https://www.omicstudio.cn/tool, and other graphs were created by GraphPad Prism. Mean ± SEM was used to plot the data. Student’s t-test, one-way ANOVA test, and two-way ANOVA test were used to analyze quantitative data between groups. A value of *p* < 0.05 was considered statistically significant.

## Supplementary Material

Supplemental Material

## Data Availability

All data supporting the findings of this study are available from the corresponding author upon request. RNA-Seq data for this article can be accessed online at https://www.ncbi.nlm.nih.gov/sra/PRJNA880980.
