# Supplementary material for: Therapeutic potential of Clostridium butyricum anticancer effects in colorectal cancer
Source: Gut Microbes. 2023 Mar 20;15(1):2186114. doi: 10.1080/19490976.2023.2186114 (PMC10038047; doi:10.1080/19490976.2023.2186114)
Supplement: Supplemental Material [file KGMI_A_2186114_SM4184.pdf]

Figure S1

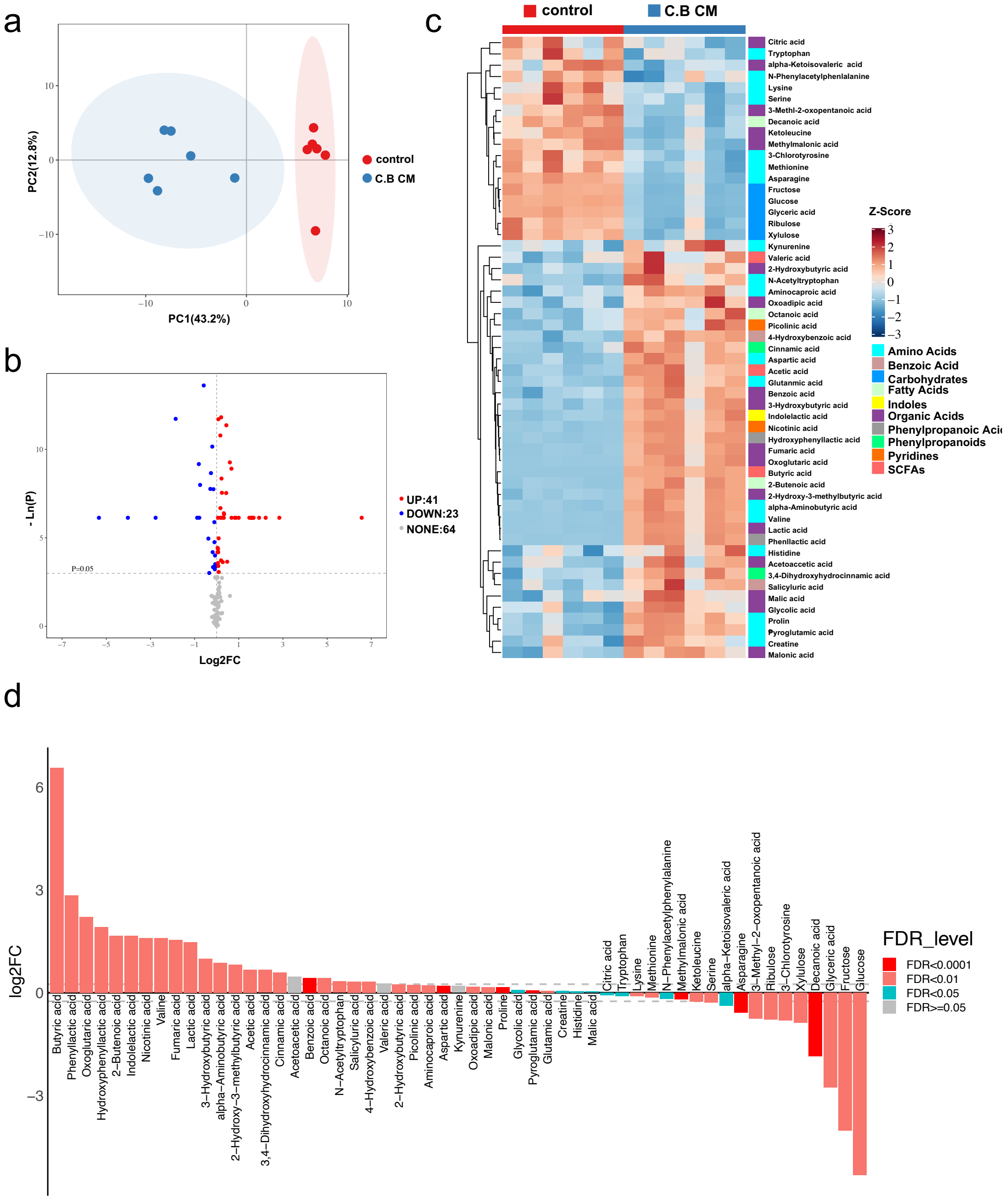
a
PC1(43.2%)
PC2(12.8%)
control
C.B CM
b
Log2FC
- Ln(P)
P=0.05
UP:41
DOWN:23
NONE:64
c
control
C.B CM
Z-Score
Amino Acids
Benzoic Acid
Carbohydrates
Fatty Acids
Indoles
Organic Acids
Phenylpropanoic Acid
Phenylpropanoids
Pyridines
SCFAs
d
log2FC
FDR_level
FDR<0.0001
FDR<0.01
FDR<0.05
FDR>=0.05

Figure S1. Metabolomics analysis of *Clostridium butyricum* conditioned medium to identify specific metabolites related to tumor inhibition.

(a) PCA 2D score plot of the supernatant from C.B cultured medium and basic cultured medium were analyzed by principal component analysis.

(b) Volcano Plot of Univariate Statistics is shown. Volcano Plot displays fold change (FC) and p value of each metabolite. In this project, threshold value for differential metabolites selection is: (1) $P < 0.05$ (2) $|log2FC| >= 0$. In volcano plot, compared with CON, differential metabolites (red highlight) in the right top corner are increased in C.B and differential metabolites (blue highlight) in the left top corner are decreased in C.B.

(c) Z-Score Plot of potential metabolite biomarkers is shown in Heat map

(d) The potential metabolite biomarkers that match the screening criteria were shown in the histogram.

Figure S2

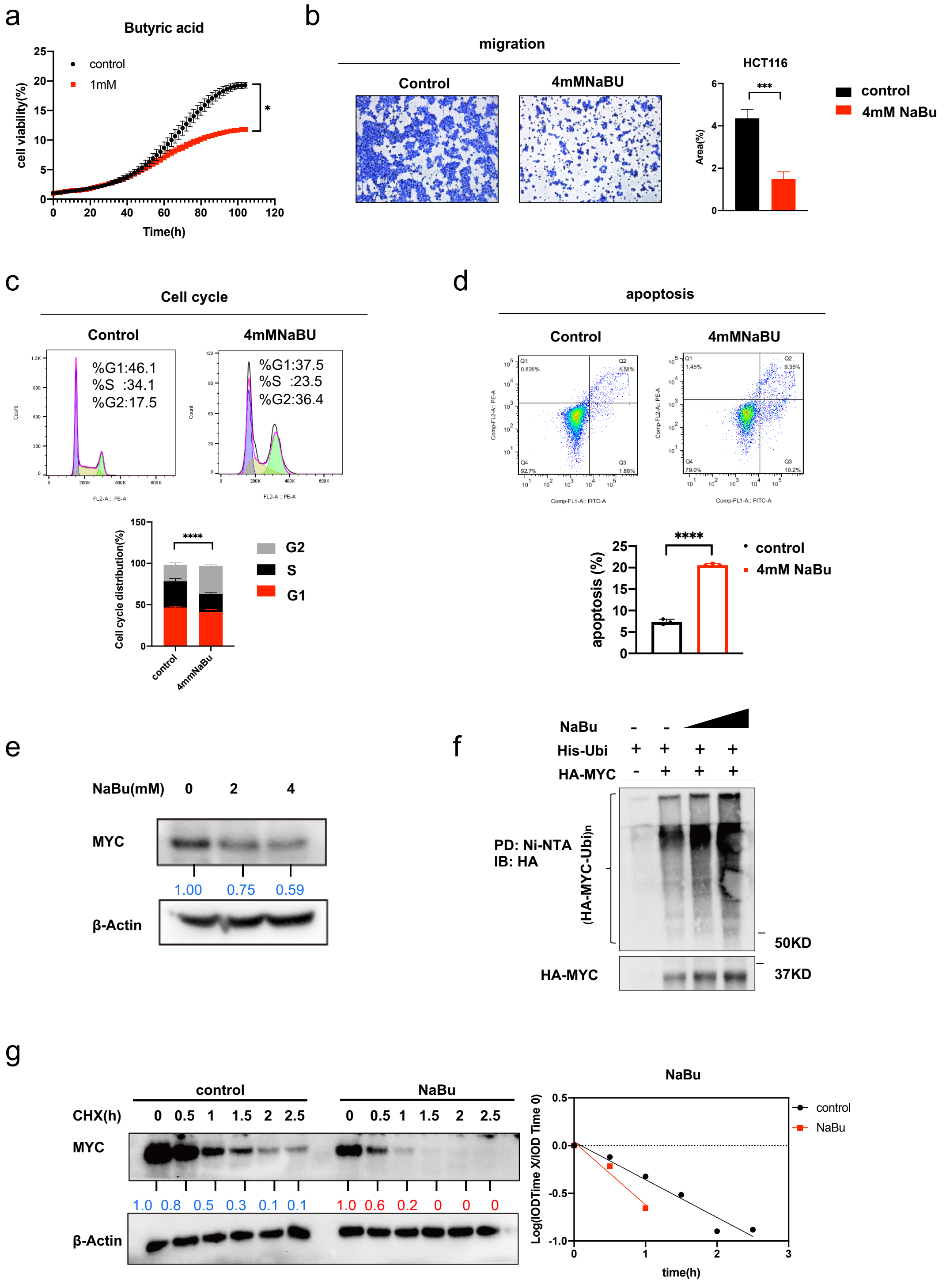
a
Butyric acid
control
1mM
cell viability(%)
Time(h)
b
migration
Control
4mMNaBU
HCT116
Area(%)
control
4mM NaBu
c
Cell cycle
Control
4mMNaBU
%G1:46.1
%S :34.1
%G2:17.5
%G1:37.5
%S :23.5
%G2:36.4
Cell cycle distribution(%)
control
4mmNaBu
G2
S
G1
d
apoptosis
Control
4mMNaBU
apoptosis (%)
control
4mM NaBu
e
NaBu(mM) 0 2 4
MYC
1.00 0.75 0.59
β-Actin
f
NaBu
His-Ubi
HA-MYC
PD: Ni-NTA
IB: HA
(HA-MYC-Ubi)n
50KD
HA-MYC
37KD
g
control
NaBu
CHX(h) 0 0.5 1 1.5 2 2.5 0 0.5 1 1.5 2 2.5
MYC
1.0 0.8 0.5 0.3 0.1 0.1
1.0 0.6 0.2 0 0 0
β-Actin
NaBu
Log(IODTime X/IOD Time 0)
time(h)
control
NaBu

Figure S2. Butyrate is effective in regulating important biological functions

(a) The growth curves of HCT116 cell treated with or without 4mM sodium butyrate (NaBU). Growth were measured by IncuCyte machine.

(b) The cell migration of HCT116 cell treated with or without 4mM sodium butyrate (NaBU). Transwell assays show the effects of treatment.

(c) Flow cytometry analysis of cell cycle distribution of HCT116 upon 4mM NaBU treatment. Cell cycle distribution (DNA histogram) in cells treated with or without NaBu for 24 h was analyzed.

(d) Flow cytometry analysis of apoptotic cells in HCT116 treated with or without 4mM NaBu for 24 h. Annexin V staining was performed.

(e) Immunoblot analysis of steady-state expression of MYC protein in HCT116 cell treated with different concentrations of NaBu (2mM and 4mM).

(f) Cells co-transfected with His-Ubi and HA-MYC plasmids were treated with NaBU. MG132 was added to the cells 6h before they were harvested. The cell lysates were pulled down with Ni-NTA beads and immunoblotted with anti-HA antibody.

(g) The turnover rate of the MYC protein in HCT116 treated with or without 4mM NaBu for 24h. CHX: cycloheximide, IOD: integrated optical density.

*, $p < 0.05$; **, $p < 0.01$; ***, $p < 0.001$; paired two-tailed Student test.

Figure S3

a

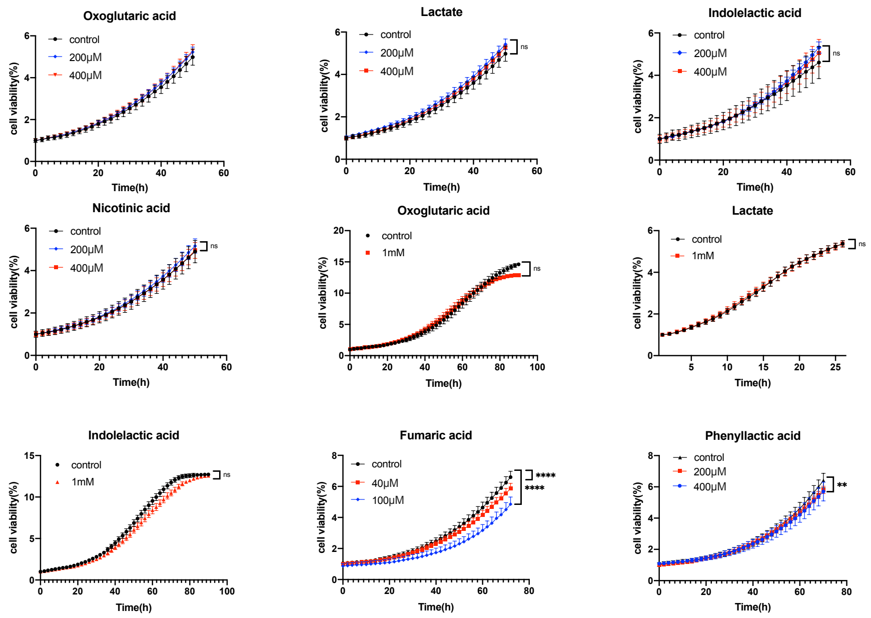
Oxoglutaric acid
control
200μM
400μM
cell viability(%)
Time(h)
Lactate
control
200μM
400μM
ns
cell viability(%)
Time(h)
Indolelactic acid
control
200μM
400μM
ns
cell viability(%)
Time(h)
Nicotinic acid
control
200μM
400μM
ns
cell viability(%)
Time(h)
Oxoglutaric acid
control
1mM
ns
cell viability(%)
Time(h)
Lactate
control
1mM
ns
cell viability(%)
Time(h)
Indolelactic acid
control
1mM
ns
cell viability(%)
Time(h)
Fumaric acid
control
40μM
100μM
****
****
cell viability(%)
Time(h)
Phenyllactic acid
control
200μM
400μM
**
cell viability(%)
Time(h)

b

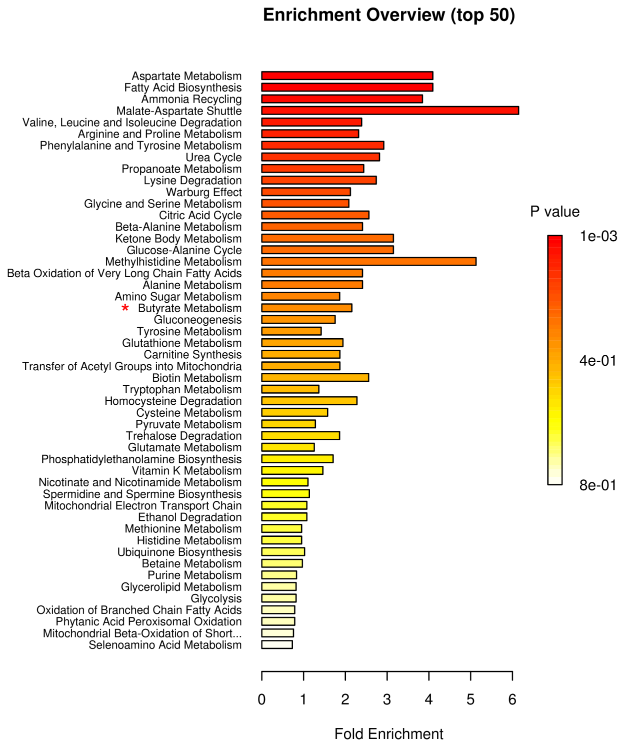
Enrichment Overview (top 50)
Aspartate Metabolism
Fatty Acid Biosynthesis
Ammonia Recycling
Malate-Aspartate Shuttle
Valine, Leucine and Isoleucine Degradation
Arginine and Proline Metabolism
Phenylalanine and Tyrosine Metabolism
Urea Cycle
Propanoate Metabolism
Lysine Degradation
Warburg Effect
Glycine and Serine Metabolism
Citric Acid Cycle
Beta-Alanine Metabolism
Ketone Body Metabolism
Glucose-Alanine Cycle
Methylhistidine Metabolism
Beta Oxidation of Very Long Chain Fatty Acids
Alanine Metabolism
Amino Sugar Metabolism
* Butyrate Metabolism
Gluconeogenesis
Tyrosine Metabolism
Glutathione Metabolism
Carnitine Synthesis
Transfer of Acetyl Groups into Mitochondria
Biotin Metabolism
Tryptophan Metabolism
Homocysteine Degradation
Cysteine Metabolism
Pyruvate Metabolism
Trehalose Degradation
Glutamate Metabolism
Phosphatidylethanolamine Biosynthesis
Vitamin K Metabolism
Nicotinate and Nicotinamide Metabolism
Spermidine and Spermine Biosynthesis
Mitochondrial Electron Transport Chain
Ethanol Degradation
Methionine Metabolism
Histidine Metabolism
Ubiquinone Biosynthesis
Betaine Metabolism
Purine Metabolism
Glycerolipid Metabolism
Glycolysis
Oxidation of Branched Chain Fatty Acids
Phytanic Acid Peroxisomal Oxidation
Mitochondrial Beta-Oxidation of Short...
Selenoamino Acid Metabolism
P value
1e-03
4e-01
8e-01
0 1 2 3 4 5 6
Fold Enrichment

Figure S3. Identify specific metabolites of C.B involved in tumor inhibition.

(a) The growth curves of HCT116 cells treated with different indicated concentrations (0, 200μM, 400μM) of several metabolites (log2FC≥1.5), including, oxoglutaric acid, lactate, nicotinic acid, indolelactic acid, phenyllactic acid (dissolved in DMSO) and fumaric acid (dissolved in DMSO). Cell growth was measured by IncuCyte machine. Phenyllactic acid and fumaric acid have growth inhibitory effect.

(b) Pathway enrichment analysis using Pathway-associated metabolite sets (Pathway-associated metabolite sets (SMPDB)) is shown. Butyrate metabolism was indicated. Data are presented as mean ± SEM. ns, no significance; *, $p < 0.05$; **, $p < 0.01$; ***, $p < 0.001$; two-way ANOVA test.

Table S1. Primers for qPCR

Gene	Forward primer sequence	Reverse primer sequence
MYC	CACCAGCAGCGACTCTGA	GATCCAGACTCTGACCTTTTGC
TYMS	TGCGCTTGGAATCCAAGAG	GATGTTGAAAGGCACACCGA
ChIP	CGCCGAGCAGGAAGAGGCGGAG	GCGGAGGATGTGTTGGATCTGC
NOTCH1	GAGGCGTGGCAGACTATGC	CTTGTACTCCGTCAGCGTGA
CD133	AGTCGGAAACTGGCAGATAGC	GGTAGTGTTGTACTGGGCCAAT
CD44	CTGCCGCTTTGCAGGTGTA	CATTGTGGGCAAGGTGCTATT
SOX9	AGCGAACGCACATCAAGAC	CTGTAGGCGATCTGTTGGGG
EPCAM	AATCGTCAATGCCAGTGTACTT	TCTCATCGCAGTCAGGATCATAA
VEGF	AGGGCAGAATCATCACGAAGT	AGGGTCTCGATTGGATGGCA
CDK4	TCAGCACAGTTCGTGAGGTG	GTCCATCAGCCGGACAACAT
1433sigma	TGACGACAAGAAGCGCATCAT	GTAGTGGAAGACGGAAAAGTTCA

BMP4	AAAGTCGCCGAGATTCAGGG	GACGGCACTCTTGCTAGGC
CDK2	CCAGGAGTTACTTCTATGCCTGA	TTCATCCAGGGGAGGTACAAC
E2F3	AGAAAGCGGTCATCAGTACCT	TGGACTTCGTAGTGCAGCTCT
GADD45A	GAGAGCAGAAGACCGAAAGGA	CACAACACCACGTTATCGGG
GADD45B	TACGAGTCGGCCAAGTTGATG	GGATGAGCGTGAAGTGGATTT
P21	CGATGGAACTTCGACTTTGTCA	GCACAAGGGTACAAGACAGTG
CYCLINE D1	TGGAGCCCGTGAAAAAGAGC	TCTCCTTCATCTTAGAGGCCAC
